# Investigating Associations between Horse Hoof Conformation and Presence of Lameness

**DOI:** 10.3390/ani14182697

**Published:** 2024-09-17

**Authors:** Fernando Mata, Inês Franca, José Araújo, Gustavo Paixão, Kirsty Lesniak, Joaquim Lima Cerqueira

**Affiliations:** 1 Center for Research in Agrifood Systems and Sustainability, Instituto Politécnico de Viana do Castelo, 4900-347 Viana do Castelo, Portugal; pedropi@esa.ipvc.pt (J.A.); gustavopaixao@esa.ipvc.pt (G.P.); cerqueira@esa.ipvc.pt (J.L.C.); 2 Escola Superior Agrária, Instituto Politécnico de Viana do Castelo, 4900-347 Viana do Castelo, Portugal; inesfranca.enfvet@gmail.com; 3 Mountain Research Centre, Instituto Politécnico de Viana do Castelo, 4900-347 Viana do Castelo, Portugal; 4 Veterinary and Animal Research Centre, Universidade de Trás-os-Montes e Alto Douro, 5000-801 Vila Real, Portugal; 5 Equine Science Department, Hartpury University, Gloucester GL19 3BE, UK; kirsty.lesniak@hartpury.ac.uk

**Keywords:** coronet band circumference, dorsal hoof wall angle, hoof–pastern axis, horses, limping

## Abstract

**Simple Summary:**

Hoof shape can provide a visual indication of imbalanced forces being transferred through the limb; such imbalances are commonly associated with lameness. The circumference of the coronet band and the angle of the hoof wall at the toe region were measured in all four feet of 73 adult horses. The horses were categorised according to their regular work requirements as either show jumpers, dressage horses, or riding centre horses. The horses were observed in their regular work by a veterinary nurse who recorded the presence or absence of lameness. For both the show jumping and the dressage horses, having either a small coronet band circumference with a large hoof wall angle or a large coronet band circumference with a small hoof wall angle increased the probability of the horse presenting with lameness in the respective limb. A small coronet band circumference with a large hoof wall angle would imply a more upright foot, which is known to be predisposed to increased concussive forces. A large coronet band circumference with a small hoof wall angle implies a broken-back hoof–pastern axis and increased loading on the suspensory apparatus.

**Abstract:**

Hoof trimming and shoeing determine the horse’s hoof shape and balance. Hoof conformation plays a crucial role in limb biomechanics and can consequently prevent or predispose to injury. This study investigated the relationship between the morphometric characteristics of the horse’s hoof, specifically, the dorsal hoof wall angle (DHWA), the coronet band circumference (CBC), and lameness in 73 horses categorised as undertaking either show jumping, dressage, or riding school activities. Results from logistic regression indicated that horses with either a combination of acute DHWA with large CBC, or more upright feet with larger DHWA and smaller CBC have higher probabilities of lameness. Show jumping and dressage horses showed a higher prevalence of lameness. Hoof morphometry should be monitored, and podiatric interventions should be regularly scheduled for the maintenance of correct hoof conformation to prevent injury. This study suggests that an aligned hoof–pastern axis managed by a DHWA of around 50 degrees may prevent lameness, with special emphasis on horses in dressage and show jumping activities. On the other hand, we can also speculate that the disturbed axis alignment of DHWA may be a cause of lameness.

## 1. Introduction

**Figure 1:**
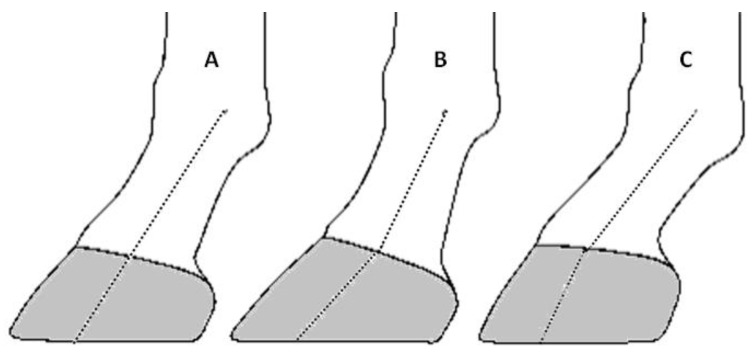
The distal limb of the horse in sagittal view: (**A**) correct hoof–pastern axis; (**B**) broken-back hoof–pastern axis; (**C**) broken-forward hoof–pastern axis. (Source: the authors).

Positioned obliquely in relation to the limb, the horse’s hoof has evolved with structural stiffness to enable the horse’s body weight to be transferred to the ground whilst being resistant to fracture [1,2]. The stiffness of the hoof capsule also serves to protect the softer tissues encapsulated within it, including the distal phalanx, the navicular bone and its associated suspensory ligaments, and the deep digital flexor tendon—all part of the equine locomotor apparatus [1]. As a result of ground contact, the distal margin of the hoof wall is subject to wear [3]. Variations in growth and wear patterns determine the conformation of the hoof and influence the mechanical loads experienced through the entire limb during locomotion [4]. Whilst wear and growth are continuous, their rates depend on factors such as the type of surfaces interacting with the hoof, and the nutritional balance of the horse’s diet [5]. Consequentially, discrepancies in the rate of wear and growth influence the angle formed between the hoof wall and the ground [4], referred to as the dorsal hoof wall angle (DHWA) [1]. For the domesticated horse, hoof care interventions, such as trimming and shoeing, significantly contribute to the hoof’s shape and balance, and play a crucial role in equine limb biomechanics [6] to prevent injury or to function therapeutically in conditions such as laminitis [7]. Hoof conformation has a well-recognised association with the risk of musculoskeletal injury [8,9,10], therefore fuelling interest in relation to its role in the management of horse health and welfare [11]. Previous investigations of hoof conformation have involved analysing parameters associated with injury risk, such as the coronet band circumference (CBC) or width (CBW), hoof–pastern axis (HPA), DHWA, and palmar/plantar angle of the distal phalanx (PADP) [9,11,12]. HPA is represented by an imaginary line that illustrates the relationship between the hoof and the pastern in the sagittal plane (Figure 1). An HPA (Figure 1A) is considered ideal when the dorsal hoof wall is parallel to the alignment of the phalanges [13,14]. As a well-recognised parameter, HPA has long been seen as an objective for farriers and veterinarians while trimming the horse’s hoof [15,16,17]. When perfectly aligned, the angle formed between the solar surface of the distal phalanx and the horizontal (the PADP) should approximate 5° [12], with a range between 2° and 10° [18].

A broken HPA refers to a lack of linearity or a broken imaginary line (Figure 1), whereby the pastern adopts a more vertical orientation in relation to the hoof, resulting in a higher positioned metacarpophalangeal joint. A broken-back HPA is often derived as a result of a long-toe and low-heel conformation. Conversely, where the heel is too long in relation to the toe, the horse presents with a broken-forward HPA, and the metacarpophalangeal joint is lowered [16,19]. Whilst a conformation issue of the foot, the misalignment of HPA affects the more proximally positioned limb joints, namely the metacarpophalangeal, the humeroradial, and the scapulohumeral joints [19]. The ideal alignment of HPA optimises not only the forces acting directly on the distal limb, but also those radiating through the proximal limb [15], thereby reducing the injury risk in the horse limb [4,16].

The physical structure and conformation of horses is shaped by both natural processes and the choices made by breeders, with the characteristics assessed for each breed depending on specific breeding objectives [20]. Achieving conformation standards are requirements in horse breeds for their admission in the breed studbook [21]. Therefore, genetics also plays a role in hoof conformation. In the literature in Section 4, it was found while determining the heritability of hoof conformation traits that the hoof–pastern axis and the pastern bone circumference are correlated with horse hoof shape. Larger bone circumferences, associated with a broader hoof shape, are linked to poorer limb conformation.

The objective of this study was to investigate the relationship of CBC and DHWA with limb lameness in horses, with the ultimate aim of finding a helpful and scientifically sound tool to identify the risk of lameness through the presence of hoof misshape. While DHWA is directly related to HPA alignment, the same does not apply to CBC. Therefore, there is interest in studying these two variables as they indirectly involve HPA alignment.

## 2. Materials and Methods

This study followed the principles of the Declaration of Helsinki. The measurement of the horses’ hooves did not raise any animal welfare concerns.

### 2.1. Data Collection

Data were collected between January and May 2022 at an equine centre (Clube Hipico do Norte) in Portugal from a random sample of 73 mature horses. All the observations and measurements were performed once on the same day for the respective horse. All the horses were stabled, had occasional access to a paddock, and undertook work at the club involving only one of the following activities: dressage, jumping, or riding school. The horses were from two origins: Portuguese (which included Lusitano and Lusitano crosses) (*n* = 36), and North European breeds (including English and Thoroughbreds) (*n* = 37). The horses were also allocated to their ‘Activity’ (riding school *n* = 28, show jumping *n* = 20, and dressage *n* = 25).

Coronet band circumference (CBC) and dorsal hoof wall angle (DHWA) were collected from all four limbs of each horse. The measurements were taken on a flat, rubber-covered surface, with the horses standing in a neutral stance, i.e., not canted-in or splayed out. CBC was measured using a flexible measuring tape. DHWA was measured directly from a lateral view of the hoof with the ‘Measure’ application on an iPhone 13 Pro, by placing the bottom of the phone on the dorsal hoof wall. The result was then subtracted from 90° to obtain DHWA. The verticality of the iPhone was verified on a flat surface and checked for horizontality with a bubble level. While placing the bottom of the iPhone on the flat surface, a reading of 0° was obtained, and while placing the iPhone on its side, a 90° reading was obtained. Limb lameness evaluations were undertaken by an experienced veterinary nurse qualified to MSc level, with certification in biomechanics obtained from the Fédération Equestre Internationale, and 10 years of practice within the equine industry.

The horses were observed in their daily activities: in their stables, standing outside, walking (ridden and unridden), ridden in trot, ridden in gallop, and while practising their regular activities. Lameness was evaluated while observing the horses ridden and unridden, walking towards and away from the observer, turning left and right, in a packed sand ground, and after warming up by being ridden for a minimum of 15 min. Then, they were also observed in trot, again ridden and unridden, walking towards and away from the observer, and turning left and right. Finally, the same procedure was implemented at a gallop. Some observations are difficult to evaluate while walking; however, they become more evident at a trot. Therefore, we assumed the presence of lameness only when perceptible at a trot, according to point two of the scale published by the American Association of Equine Practitioners [22].

Lameness was registered as present or absent for each of the respective limbs (front left, front right, hind left, and hind right).

### 2.2. Statistical Methods

Using the presence/absence of lameness as a dependent variable, and the horse as the unit of analysis, the fitness of several generalised linear models from the binomial family were tested, using a diverse range of link functions. The independent variables considered were the factors ‘Activity’ (levels: dressage, show jumping, and riding school), ‘Breed’ (levels: Portuguese and North European), ‘Hoof’ (left front, left hind, right front, and right hind), and the covariables ‘DHWA’ and ‘CBC’.

As the covariate measurements were obtained for each of the horses’ four limbs, a set of *n* = 73 × 4 = 292 data entries was obtained. 

As we have four observations (one per limb) per horse while using the horse as the unit of analysis, we verified the existence of paired data for the factor ‘Hoof’. As such, we started our analysis using a generalised estimating equations model. Despite documented differences in biomechanics and hoof shape [23,24] between the fore and the hindlimb, the factor ‘Hoof’ was not found to be significant after testing the model, and therefore, the models tested thereafter were generalised linear models for independent data, without further consideration given to the factor ‘Hoof’, thus resulting in the limb being used as the unit of analysis.

The tested models were factorial. We used a stepwise backward procedure for the selection of significant variables and/or interactions, using Wald’s chi-square test. The level of significance was set at *p* < 0.05. 

The model adjustment was evaluated through the likelihood ratio chi-square. The comparison of the adjustment of the different models was made using Akaike’s Information Criterion (AIC).

Independent samples t-tests were performed to assess eventual significant differences in lame and healthy horses, concerning the variables CBC and DHWA.

The statistical analysis was performed through the software IBM Corp.^®^ SPSS^®^ Statistics, Armonk, NY, USA. Version: 29.0.2.0 (20).

## 3. Results

Descriptive statistics for CBC and DHWA measurements can be viewed in Table 1.

No significant differences (*p* > 0.05) were determined between the presence and absence of lameness in relation to the variables CBC and DHWA. Table 2 reports the percentage of horses with lameness and its distribution according to the number of lame limbs. Table 3 reports which horse limbs were identified with lameness.

The most parsimonious model was achieved with a logit link; therefore, a logistic regression was fitted to the data (likelihood ratio χ^2^ = 188,53; 10 df; *p* < 0.001), AIC = 222.98. The parameters of the adjusted model are outlined in Table 4. The variable ‘Breed’ was not found to be significant in the model (*p* > 0.05).

The parameters identified in Table 4 were entered into the generic Equation (1) to calculate the probability of the presence of lameness in the limb of a given horse.

πc = 1/(1 + exp (*β*_1_X_1_ × *β*_2_X_2_ + *β*_3_X_3_ × *β*_4_X_1_ + *β*_5_X_1_ × *β*_6_X_2_X_3_)) (1)

where: 

*β*_1_ is the parameter associated with ‘Activity’, and X_1_ the respective dummy variable;

*β*_2_ is the parameter associated with CBC, and X_2_ the respective CBC value;

*β*_3_ is the parameter associated with DHWA, and X_3_ the respective DHWA value;

*β*_4_ is the parameter associated with the interaction between ‘Activity’ and CBC;

*β*_5_ is the parameter associated with the interaction between ‘Activity’ and DHWA.

The probability of the presence of lameness in the limb of a given horse is, therefore, calculated by Equation (2).

1 − πc = 1 − 1/(1+exp (*β*_1_X_1_ × *β*_2_X_2_ + *β*_3_X_3_ × *β*_4_X_1_ + *β*_5_X_1_ × *β*_6_X_2_X_3_)) (2)

**Figure 2:**
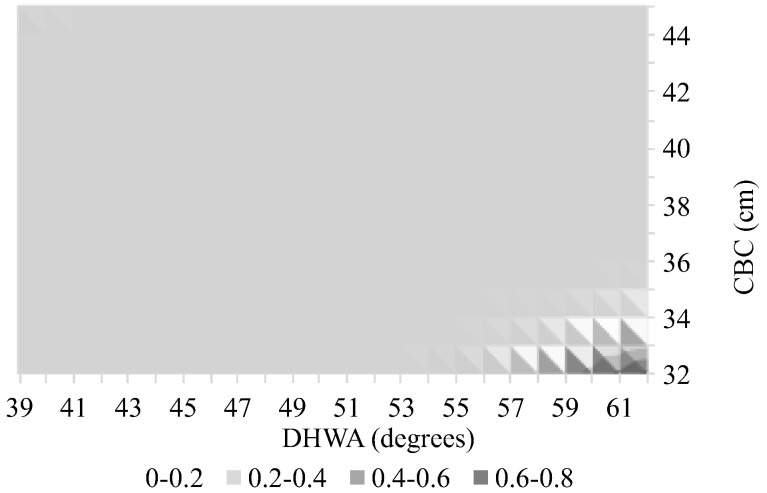
Probability of the presence of lameness in horses undertaking riding school activity, as a function of the coronet band circumference (CBC) and the dorsal hoof wall angle (DHWA).

**Figure 3:**
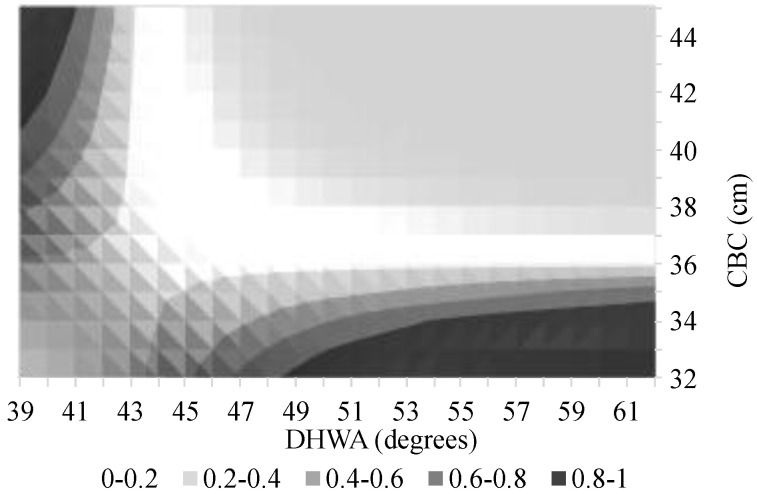
Probability of the presence of lameness in horses undertaking dressage activity, as a function of the coronet band circumference (CBC) and the dorsal hoof wall angle (DHWA).

**Figure 4:**
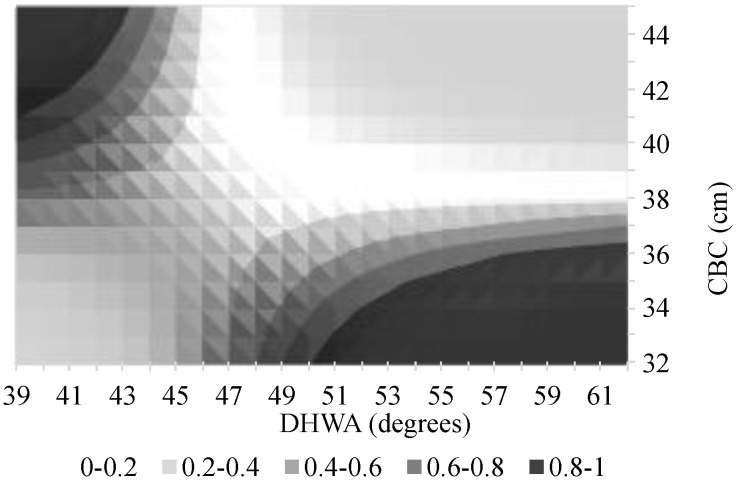
Probability of the presence of lameness in horses undertaking show jumping activity, as a function of the coronet band circumference (CBC) and the dorsal hoof wall angle (DHWA).

Figure 2, Figure 3 and Figure 4 are the graphical representations of the model with probabilities of lameness calculated for the different ‘Activities’ while varying the covariates in the values indicated in the figures’ axes.

In the graphs of Figure 3 and Figure 4, we can observe that larger CBC associated with smaller DHWA and vice versa lead to higher probabilities of lameness identification. The graph of Figure 2 shows the same tendency but for larger DHWA associated with smaller CBC only.

## 4. Discussion

In our sample, we have identified 39 out of 73 horses with lameness (54.1%), which agrees with the results of other studies. In a study in Switzerland [25], the authors report that 54.4% of the horses observed were classified as being lame after a score of at least 2 out of 5 on the scale reported by Weishaupt et al. [26,27]. Another author [28], also in Switzerland, reported lameness incidences of 49%. Of the 39 horses that we identified with lameness, 22 were lame in one limb only (56.4% of the horses), 14 in two limbs (35.9%), 3 in three limbs (7.7%), and no horses presented lameness in all four limbs. Hibbs et al. [29] reported 7.1%, 44,8%, 22.9%, and 24.2%, respectively, from a sample of 520 lame horses.

The current study aimed to determine whether a relationship could be identified between hoof conformation parameters, specifically CBC and DHWA, and the presence, or absence, of lameness in the working horse. The range (39° to 62°) and mean values with a 95% confidence interval (48.8°, 48.3°, and 49.3°) for DHWA in the current study are similar to those previously reported in the literature, which range from 42° to 58° [30,31] with a mean value between 47.7 [30] and 53.7° [31]. The CBC range (32.2 cm to 45 cm) and mean values with a 95% confidence interval (38.3 cm, 38.01 cm, and 38.59 cm) are also in agreement with previously reported values (range = 21.6 to 56.9; mean = 39.2) [9].

Research has recognised the importance of correct DHWA in limb joint health. Rooney [13] reports that DHWA directly affects the pastern and coffin joints and inversely affects the fetlock. An abnormal DHWA results in misalignment of the phalanges [32], and by default, negatively impacts the positioning of the proximal and distal interphalangeal joints and the metacarpophalangeal joint [19]. A broken-back hoof–pastern axis leads to the dorsiflexion of the coffin joint, concentrating weight on the foot’s palmar section and increasing strain on the deep digital flexor tendon, which can heighten stress on the navicular apparatus and the soft tissue structures associated with the navicular bone. When causing heel pain, the horse may land toe first, potentially resulting in subsolar bruising. This abnormal hoof conformation can contribute to palmar foot pain, chronic heel bruising, coffin joint synovitis, quarter and heel cracks, and interference issues [32]. Poor performance and injuries linked to a broken-forward hoof–pastern axis are believed to involve coffin joint inflammation due to abnormal joint loading, sole bruising, and increased strain on the suspensory ligaments of the navicular bone [32].

Sustained increases in tension will lead to microdamage and pain. Lameness would subsequently ensue as the musculoskeletal system adjusts to minimise the effect of the imbalanced loading [33]. Feet with an acute wall angle, such as those with a broken-back HPA, have significantly increased time to breakover and subsequently increased tensile loads on the DDFT [34]. Furthermore, acutely angled hooves are more likely to strike the ground with a toe-first impact [34], which is a characteristic also associated with lame horses [35,36].

The coronet band circumference is reported to vary greatly between horses, a finding mainly attributed to differences in body weight, which was not a variable in our study. Turner [37] reported that hoof size as a function of body mass should not exceed 5.5 kg/cm^2^, with more weight per square centimetre highly likely to lead to lameness. CB dimensions are reported to be influenced by loading factors. In horses above 1.63 m in height, Lesniak [9] concluded that increases in mass increased the width of the base of the hoof, without a proportional increase in CB width. Given that horses greater in height (>1.7 m vs <1.63 m) have an increased risk (15%) of lameness [38], and greater body masses have been associated with hoof disorders such as laminitis [38,39,40,41], this is an important point to consider in terms of load distribution. Furthermore, CBC has been reported to vary depending on training regimes, with decreases observed as training intensity progresses and reversed when training is halted [42]. Whilst interaction between variables makes the results from the current study challenging to interpret, both the acute DHWA with large CBC and the larger DHWA with reduced CBC result in a greater probability of lameness.

The combination of small DHWA with larger CBC modelled from the results of this study could reflect increases in the tensile loading of the deep flexor tendon and the interosseous medium suspensory ligament. Although DHWA does not always reflect the angle of the solar margin of the distal phalanx, acute DHWA is associated with long-toe and low-heel conformation, which is known to significantly increase the breakover duration and the maximal extension of the deep digital flexor tendon [43].

Results from the current study indicated a strong association between hoof conformation and lameness, particularly within those horses used predominantly for dressage and show jumping activities. This finding aligns with those of Sousa et al. [44], who found that in horses dedicated to activities such as show jumping and dressage, which require longer training, joint diseases are more predominant.

The lateral movements commonly observed in dressage are responsible for an atypical strain on the horse’s back and impose additional twisting of the appendicular joints [45]. Conversely, significant stresses are applied to the suspensory apparatus of the showjumper in the hind limb on take-off and in the forelimb on landing. When considered alongside the substantial torque applied to joints while turning, [45] this creates a potential for lameness when the horse is also subject to an incorrectly proportioned hoof conformation.

Appropriate hoof care in foals is crucial for their future athletic potential. The proper management of foal feet and limbs during early life influences their development. Understanding hoof development and shape is essential to avoid harm from excessive or misguided treatments [46]. Foals with imbalances should have their hooves trimmed from a young age to eliminate imbalances. While studying the prevalence of uneven feet, Ducro et al. [47] found that these increase with age in both males and females. Uneven feet have been found to be more prevalent in lame than in sound horses [48].

As a limitation of this study, we would like to highlight that only one person evaluated lameness a single time. Lameness is a complex issue that may result from various reasons that are not completely understood [49,50,51], and a collegial evaluation using a grading system could eventually be more accurate and provide an additional covariate for evaluation (degree of lameness). Despite the reasonable number of horses used in this study, we acknowledge that a larger sample of horses, after power analysis, from a variety of settings could also increase reliability. Further studies should address the limitations herein identified and eventually add other variables such as horse weight.

## 5. Conclusions

The relationship between the morphometric characteristics of the horse’s hoof (DHWA and CBC) and the presence of lameness was investigated. These morphometric characteristics are easily obtained and may be indicative of a predisposition to lameness, or indeed reflective of an existing sub-clinical lameness. It is therefore paramount to manage these aspects of the horse hoof to prevent lameness either occurring or progressing. As practices which manipulate DHWA and the heel angle, and therefore HPA, equine podiatry and farriery are paramount in the detection and early management of lameness. Strategies such as corrective foot trimming or remedial shoeing can be used to assist this process. The horse owner also needs to be able to identify abnormal characteristics and seek timely podiatric interventions to prevent imbalanced loading and the potential for injury. A correct hoof shape, achieved through preventive podiatric interventions, can provide health and welfare to horses, thereby preventing expensive curative measures that could also disrupt their normal activities.

## Figures and Tables

**Table 1:** Descriptive statistics for the coronet band circumference (CBC) and the dorsal hoof wall angle (DHWA), taken from the four limbs of the 73 horses sampled (*n* = 292).

	N	Minimum	Maximum	Mean	SD
CBC (cm)	292	32.2	45.0	38.3	2.50
DHWA (°)	292	39.0	62.0	48.8	4.37

**Table 2:** Number of lame horses and respective percentages.

Sample	Ner of Horses	% of Sample	% of Lame
All horses	73	100	
Not lame	34	45.9	
lame	39	54.1	100
1 limb	22		56.4
2 limbs	14		35.9
3 limbs	3		7.7
4 limbs	0		0

**Table 3:** Location of the lameness in the horses observed.

Limb	Number of Observations with Lameness
Front left and hind right	4
Front left and hind left	1
Hind left and hind right	1
Front right and hind right	4
Front left and hind left	4
Front left, front right, and hind left	1
Front left, hind left, and hind right	2

**Table 4:** Parameters of the adjusted logistic model. The absence of lameness is modelled, and the presence of lameness is the reference.

Parameter	*β*	SE	Wald 95% CI		Wald χ^2^	df	*p*-Value	OR
Activity ***								
Riding School	279.775	48.767	184.193	375.356	32.913	1	<0.001	3.19 × 10^121^
Dressage	236.208	43.221	151.497	320.919	29.868	1	<0.001	3.84 × 10^102^
Show Jumping	268.278	45.760	178.590	357.966	34.371	1	<0.001	3.25 × 10^116^
CBC ***	−7.012	1.194	−9.352	−4.673	34.507	1	<0.001	0.90 × 10^−3^
DHWA ***	−5.737	0.967	−7.632	−3.841	35.195	1	<0.001	0.32 × 10^−2^
Activity × CBC *								
Riding School × CBC	−0.173	0.222	−0.608	0.262	0.605	1	0.437 ^NS^	0.84
Dressage × CBC	0.476	0.221	0.044	0.909	4.654	1	0.031	1.61
Show Jumping × CBC	Reference							
Activity × DHWA **								
Riding School × DHWA	−0.088	0.110	−0.304	0.129	0.631	1	0.427 ^NS^	0.92
Dressage × DHWA	0.317	0.123	0.076	0.559	6.643	1	0.010	1.37
Show Jumping × DHWA	Reference							
CBC × DHWA ***	0.151	0.025	0.101	0.200	35.435	1	<0.001	1.16

Notes: SE: standard error; CI: confidence interval; df: degrees of freedom; CBC: coronet band circumference; DHWA: dorsal hoof wall angle; OR: odds ratio. Levels of significance of the variables: ^NS^ Non-significant, * *p* < 0.05, ** *p* < 0.01, *** *p* < 0.001.

## Data Availability

The raw data supporting the conclusions of this article will be made available by the authors upon request.
